# Sustainable Valorization of Palm Fatty Acid Distillate into Amide-based Corrosion Inhibitors for Carbon Steel Protection in CO_2_‑Saline Environments

**DOI:** 10.1021/acsomega.6c04652

**Published:** 2026-09-04

**Authors:** Kornravee Suwanna, Sureeporn Ruengsangtongkul, Panyawat Wangyao, Korakot Sombatmankhong

**Affiliations:** † Metallurgical Engineering Department, Faculty of Engineering, 26683 Chulalongkorn University, Phayathai Road, Bangkok 10330, Thailand; ‡ National Energy Technology Center (ENTEC), 61191 National Science and Technology Development Agency, 114 Thailand Science Park, Phahonyothin Road, Khlong Nueng, Khlong Luang, Pathum Thani 12120, Thailand

## Abstract

A PFAD-based amide corrosion inhibitor was synthesized from palm fatty acid distillate (PFAD), an abundant palm oil refining byproduct in Thailand, and diethylenetriamine (DETA) to enhance PFAD value and reduce inhibitor production costs. The synthesized amide was confirmed by ^1^H NMR, ^13^C NMR, and FTIR analyses. Its inhibition performance for X65 carbon steel in CO_2_-saturated 1 wt % NaCl solution was evaluated using LPR, PDP, and EIS at different concentrations. PDP and LPR results showed pronounced decreases in corrosion current density and corrosion rate, with the highest efficiencies of 67.09% and 71.85%, respectively, at 300 ppm, while EIS after 24 h showed a maximum efficiency of 81.97% at 500 ppm. The inhibition performance was attributed to the formation of an adsorbed protective film, demonstrating the potential of PFAD-derived amides as cost-effective and sustainable corrosion inhibitors for oil and gas pipelines.

## Graphical abstract

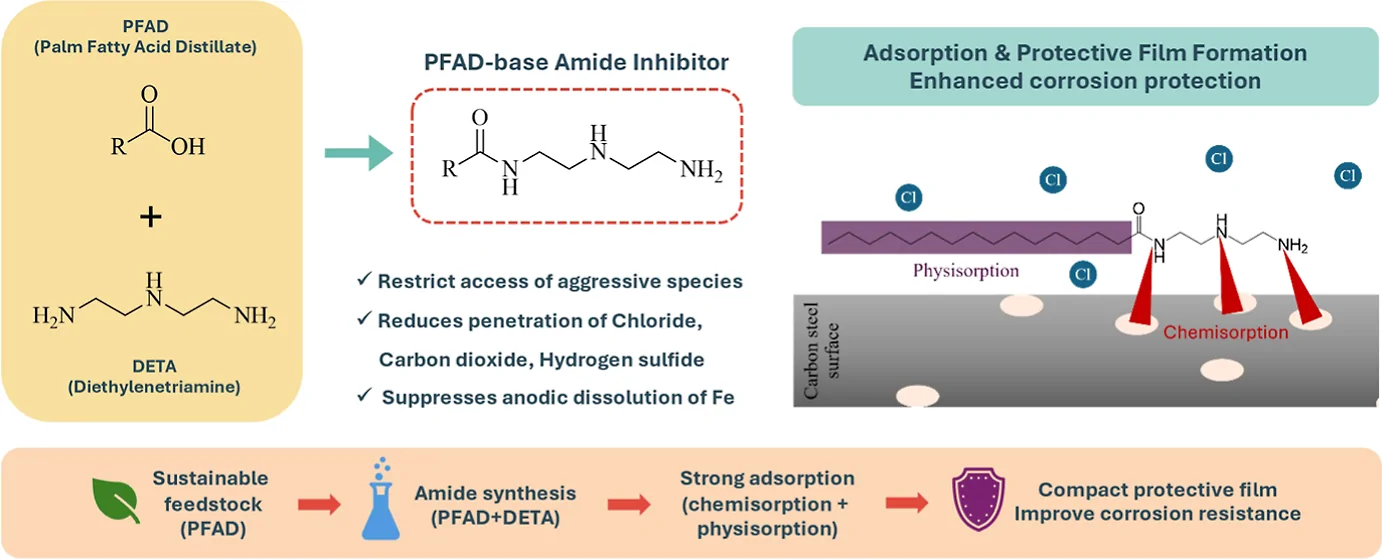

## 1 Introduction

Corrosion poses a significant challenge across various industries, particularly in oil and gas transportation. In oilfield operations, harsh service environments often lead to progressive material degradation, resulting in serious structural and economic consequences. Consequently, metallic components used throughout production stages—including drilling, refining, transportation, and storage—are highly susceptible to corrosion.^1^ In industrial manufacturing and processing, material selection for oil and gas facilities plays a crucial role in determining both operational efficiency and equipment longevity. Among the available materials, carbon steel remains the most widely used due to its cost-effectiveness and favorable mechanical properties, which make it suitable for a broad range of applications.^2,3^ However, despite these advantages, carbon steel suffers from poor corrosion resistance, necessitating effective mitigation strategies to enhance its durability, reduce maintenance costs, and improve overall process efficiency. The predominant forms of corrosion in such systems include CO_2_-induced (sweet) corrosion, H_2_S-induced (sour) corrosion,^4^ and oxygen-related corrosion from dissolved gases in injection water. Studies have shown that more than 25% of all failures in the oil and gas sector are attributed to corrosion, with CO_2_ corrosion alone accounting for approximately 28% of these cases.^5^ To mitigate corrosion-related failures, industries employ various techniques such as coatings, cathodic protection, chemical inhibitors, and routine inspection and maintenance. Although cathodic protection effectively suppresses corrosion reactions, it requires expensive installation and maintenance, leading to high operational costs.^6^ In contrast, corrosion inhibitors offer a more cost-effective, versatile, and easily applicable solution. They are often used in conjunction with cathodic protection to achieve optimal results. Nevertheless, due to their simplicity, adaptability, and economic advantages, chemical inhibitors are frequently favored over cathodic protection in oil and gas operations.

Corrosion control is essential for all industries, as it significantly impacts the integrity and lifespan of pipelines. Being constantly exposed to harsh environmental conditions, pipelines are particularly vulnerable to accelerated corrosion. Corrosion inhibitors are widely used to address this problem.^7–9^ When applied in small concentrations,^10^ these chemicals effectively reduce corrosion rates by forming adsorbed protective films that suppress the electrochemical reactions responsible for metal dissolution. Corrosion inhibitors can be classified according to their mechanism of action: anodic inhibitors protect the anodic sites, cathodic inhibitors reduce the cathodic reaction rate, and mixed-type inhibitors provide protection at both sites. In addition, inhibitors can be categorized by their chemical composition—organic inhibitors, which contain heteroatoms such as nitrogen, oxygen, or sulfur, and inorganic inhibitors, which form protective inorganic layers on metal surfaces.^11,12^

Corrosion inhibitors commonly contain various functional groups, particularly polar head groups such as fatty acids, amines, imidazolines, and amine oxides, as well as oxygen-, sulfur-, or phosphorus-containing salts, together with long hydrocarbon chains (typically C_14_–C_18_) that enhance adsorption onto metal surfaces.^13^ Tall oil fatty acid (TOFA), obtained as a byproduct of the kraft pulping process in the pulp and paper industry,^14^ is one of the most widely used sources of free fatty acids for synthesizing corrosion inhibitors. TOFA is composed mainly of oleic acid and linoleic acid, with palmitic acid as a secondary component. Alternatively, palm fatty acid distillate (PFAD)—a byproduct of palm oil refining—contains a high concentration of free fatty acids (FFA) and is extensively employed in biodiesel production, biochemical processes, soap manufacturing, and oleochemical industries. PFAD typically comprises more than 80% FFA, primarily palmitic acid and oleic acid, while the remaining fraction (∼20%) includes triglycerides, partial glycerides, vitamin E, sterols, and squalene.^15^ Both palmitic and oleic acids in PFAD are well-known precursors for the synthesis of corrosion inhibitors. Thailand holds a distinct advantage in oleochemical development, as PFAD is abundantly available domestically and serves as a low-cost, renewable feedstock for the production of biobased corrosion inhibitors.^16^ While several studies have employed crude palm oil for inhibitor synthesis, the use of PFAD as a precursor for corrosion inhibitor synthesis remains largely unexplored, presenting an opportunity for developing sustainable, palm-based corrosion inhibitors with strong adsorption capability and environmental compatibility.

Al-Edan et al.^13^ synthesized a sustainable amide-based corrosion inhibitor, *N*-(4-aminobutyl)­palmitamide (BAPA), using a green and rapid method. BAPA effectively delayed and reduced corrosion of mild steel in 1 M HCl by forming a protective surface film. The inhibition efficiency increased with concentration, reaching 91.5% at 65 ppm, and a strong correlation was observed between experimental results and the adsorption model. Surface morphology analysis confirmed the formation of a compact protective layer attributed to the interaction between BAPA molecules and the mild steel surface. Similarly, Solomon et al.^17^ reported the synthesis of a novel palmitic imidazoline compound, *N*-(2-(2-pentadecyl-4,5-dihydro-1*H*-imidazole-1-yl)­ethyl)­palmitamide (NIMP), which was characterized by ^1^H- NMR, ^13^C NMR, and FTIR spectroscopy. NIMP was evaluated as a corrosion inhibitor for N80 steel in 15% HCl under both ambient (25 °C) and elevated (60 °C) temperatures using weight loss, electrochemical, and surface analyses. The results demonstrated that NIMP effectively suppressed steel dissolution, with optimal inhibition observed at 300 ppm. Adsorption studies indicated a chemisorption mechanism, and potentiodynamic polarization (PDP) analysis revealed that NIMP acted as a mixed-type inhibitor, predominantly influencing the cathodic reaction, consistent with surface morphology observations. These findings highlight NIMP’s potential as an acidizing corrosion inhibitor. Tao et al.^18^ synthesized a nitrophenyltriazole derivative (TMANO) and investigated its performance as a corrosion inhibitor for mild steel in 0.5 M H_2_SO_4_. Inhibition behavior was assessed through weight loss, electrochemical impedance spectroscopy (EIS), potentiodynamic polarization (PDP), and scanning electron microscopy (SEM). Thermodynamic and kinetic analyses showed that TMANO adsorption followed the Langmuir isotherm, and the process was exothermic with a negative entropy change, indicating spontaneous adsorption through a well-ordered film formation. Daniyan et al.^19^ demonstrated the effectiveness of palm oil as a natural corrosion inhibitor for ductile iron and mild steel in 1 M NaOH under aerated conditions. Palm oil, rich in palmitic and oleic acids, showed high inhibition efficiency based on long-term weight loss studies. Remarkably, no significant corrosion was observed on metal samples throughout 1344 h of exposure when the inhibitor was present, confirming the strong protective capability of palm oil-derived compounds against alkaline corrosion.

Recent studies have increasingly demonstrated that materials obtained from biomass residues and waste resources can serve as sustainable corrosion-protection agents in chloride-containing and saline environments. Reported systems include copper surfaces protected by Buddhist pine leaf extract, mild steel coated with watermelon seed extract/silica nanocomposites, garlic peel extract coatings on mild steel, waste spinach leaf extract applied to copper, coatings derived from waste lady-finger caps, and cashew nut shell liquid-based protective films.^20–25^ Collectively, these works indicate that naturally derived compounds possessing heteroatoms, polar active groups, and hydrophobic segments are capable of attaching to metallic substrates and producing protective barrier films. This evidence supports the present approach of converting PFAD into an amide-based corrosion inhibitor.

Most commercially available corrosion inhibitors are synthesized using TOFA as a precursor. However, because TOFA is an imported commodity, its procurement substantially increases the overall production cost, thereby raising the price of the final inhibitor formulations. In contrast, PFAD—a low-cost byproduct of the palm oil refining process for biodiesel production—is abundantly available in Thailand but remains underutilized in high-value chemical applications. This study introduces a novel and sustainable approach by employing PFAD as a renewable and economical feedstock for synthesizing amide-based corrosion inhibitors. The work represents a first demonstration of converting Thailand’s biodiesel-derived PFAD into high-value, environmentally benign corrosion protection materials. This strategy not only reduces the dependence on imported TOFA, but also enhances the circular utilization of domestic palm oil byproducts, thereby linking green chemistry with industrial waste valorization. The synthesized amide was structurally characterized using ^1^H NMR, ^13^C NMR, and FTIR spectroscopy to confirm successful formation. Its corrosion inhibition performance on X65 carbon steel in CO_2_-saturated 1 wt % NaCl solutions was systematically evaluated at various concentrations through LPR, PDP, and EIS techniques. Furthermore, scanning electron microscope and FTIR analysis of the adsorbed film were performed to elucidate the surface morphology and adsorption mechanism. Overall, this work introduces a sustainable and cost-effective route for developing PFAD-derived amide inhibitors, contributing both to green chemistry advancement and to the valorization of Thailand’s biodiesel byproducts, marking a novel step toward economically and environmentally sustainable corrosion protection solutions.

## 2 Experimental Section

### 2.1 Chemicals

PFAD was supplied by Patum Vegetable Oil Co., Ltd. Diethylenetriamine (DETA, 99.0%) was purchased from Sigma-Aldrich. Dichloromethane (DCM, 99.8%) was obtained from Labsolv, and sodium chloride (NaCl, 99.5%) was purchased from Honeywell Fluka. Xylene (99.0%), anhydrous sodium sulfate (99.0%), and anhydrous ethyl alcohol (99.9%) were supplied by Daejung. All chemicals were of analytical grade and used without further purification.

### 2.2 Synthesis of PFAD-Based Amide

The synthetic PFAD-based amide, illustrated in Figure 1, was prepared in a 250 mL four-neck round-bottom flask equipped with a condenser, thermometer, Dean–Stark trap, and magnetic stirrer. The flask was charged with 10 g of PFAD and 5 mL of DETA, then 40 mL of xylene was added as the solvent. The mixture was heated under reflux using a heating mantle with magnetic stirring. The reaction was initiated at 140 °C and maintained for 4 h, after which the amidation reaction was continued at 170 °C overnight. The crude product was subsequently treated in a Dean–Stark apparatus at 170 °C for 1 h to remove residual solvent and water, yielding the PFAD-based amide.

**Figure 1:**
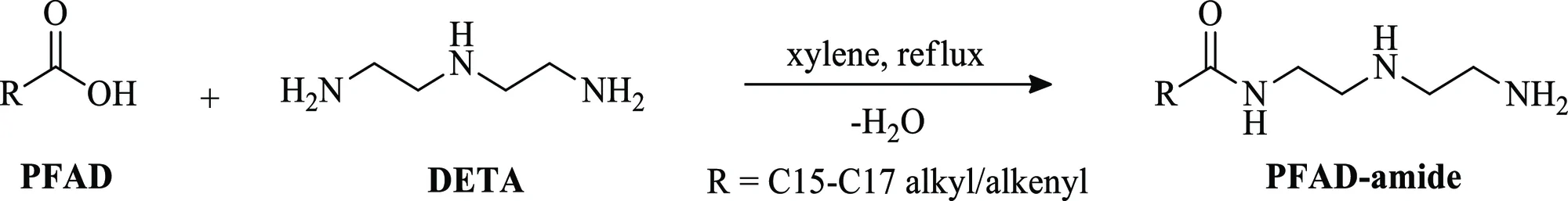
Schematic illustration of PFAD-based amide synthesis.

### 2.3 Characterization of Synthetic PFAD-Based Amide

To elucidate the molecular structure and identify the functional groups of the synthesized PFAD-based amide, spectroscopic analyses were performed using ^1^H NMR and ^13^C NMR spectrometer, and Fourier Transform Infrared (FTIR) spectroscopy. The ^1^H NMR and ^13^C NMR spectra were recorded on a Bruker 500 MHz spectrometer using CDCl_3_ as the solvent. The FTIR spectra were obtained using a Bruker Invenio R FTIR spectrometer equipped with an attenuated total reflection (ATR) accessory. Spectra were collected in the range of 4000–400 cm^–1^ in transmission mode, with 64 scans per sample to ensure optimal resolution and signal-to-noise ratio.

### 2.4 Specimens and Solution Preparation

A 5 mm diameter X65 carbon steel electrode was used as a rotating disk electrode (RDE) for polarization measurements. For the EIS experiments, a disk-shaped X65 carbon steel specimen with a diameter of 50 mm and a thickness of 2 mm was employed. Each X65 steel coupon exposed an effective surface area of 0.785 cm^2^ to the test solution. Prior to each electrochemical measurement, the specimens were wet-polished sequentially using silicon carbide (SiC) abrasive papers of 1000, 1500, and 2000 grit, followed by rinsing with ethyl alcohol and air drying. The corrosion inhibitor solution was prepared by dissolving 70 mg of the synthesized PFAD-based amide in a mixed solvent system containing 6.67 μL of xylene and 3.33 μL of ethanol, ensuring homogeneous dispersion of the inhibitor prior to testing.

### 2.5 Corrosion Test

Electrochemical tests, including linear polarization resistance (LPR) and EIS, were performed using a Metrohm Autolab PGSTAT204 Potentiostat/Galvanostat in a CO_2_-saturated 1 wt % NaCl solution at ambient temperature. Measurements were conducted in both the absence of inhibitor (0 ppm) and in the presence of the PFAD-based amide at concentrations of 100, 200, 300, 400, and 500 ppm. A standard three-electrode glass cell was employed, consisting of a platinum (Pt) rod as the counter electrode, a silver/silver chloride (Ag/AgCl) electrode as the reference, and an X65 carbon steel specimen as the working electrode. This setup was used to monitor the open-circuit potential (OCP) and to perform polarization and impedance studies. The OCP was recorded for 24 h to ensure system stabilization prior to measurement. Polarization tests were carried out within a potential range of −20 to +20 mV relative to OCP, at a scan rate of 0.125 mV s^–1^, and repeated every 40 min over a 6 h period. The polarization resistance (*R*_p_) was determined from the slope of the potential–current curve near the corrosion potential (*E*_corr_). For EIS measurements, the frequency range was set between 100 kHz and 0.1 Hz. The inhibition efficiency (η %) was calculated based on data obtained from the PDP, LPR, and EIS measurements using eqs 1–4, respectively.

1 $$\eta_{PDP}=\left(1-\frac{I_{corr}}{I_{corr}^{0}}\right)x\;100$$

2 $$\eta_{LPR}=\left(1-\frac{R_{p}^{0}}{R_{p}}\right)x\;100$$

3 $$\eta_{EIS}=\left(1-\frac{R_{p}^{0}}{R_{p}}\right)x\;100$$

4 $$CR\;\left(mmpy\right)=3.27\times 10^{‐3}\times \left(I_{corr}\times EW/\rho \right)$$

where *I*_corr_ and *I*_corr_^0^ are represent the corrosion current densities in the presence and absence of the corrosion inhibitor, respectively. Likewise, *R*_p_ and *R*_p_^0^ denote the total polarization resistance of the X65 carbon steel specimen in the test solution with and without the inhibitor, respectively. EW is the equivalent weight of the steel; and ρ is the density of the steel.

### 2.6 Surface Examination

The surface morphology of the X65 carbon steel specimens was examined after immersion in a 1 wt % NaCl (brine) solution under both inhibited and uninhibited conditions for 24 h. Surface imaging was performed using a scanning electron microscopy (SEM) to observed morphological changes, assess the formation and coverage of protective films, and detect signs of corrosion or pitting on the metal surface.

Additionally, FTIR spectroscopy was employed to identify the functional groups associated with the adsorbed corrosion inhibitor film, providing insights into the adsorption behavior and interaction mechanism between the PFAD-based amide and the steel surface.

## 3 Results and Discussion

### 3.1 Characterization of Synthetic Corrosion Inhibitors

The formation of a synthetic PFAD-based amide inhibitor from PFAD and DETA was verified using ^1^H NMR, ^13^C NMR, and FTIR analysis, as shown in Figures 2 and 3. The molecular weight of the synthetic PFAD-based amide was evaluated by high-resolution mass spectrometry (HRMS) to confirm the structure of the compound, which was determined to be 341.34 g/mol. The successful synthesis of the synthetic PFAD-based amide was confirmed by the ^1^H NMR (Figure 2a) and ^13^C NMR (Figure 2b) results. The following NMR peaks were observed; ^1^H NMR δ: 0.85 (3H, t, *J* = 6.96 Hz), 1.24 (24H, q, *J* = 9.07 Hz), 1.57 (2H, d, *J* = 6.89 Hz), 1.97 (1H, q, *J* = 6.37 Hz), 2.15 (2H, t, *J* = 7.72 Hz), 2.75 (2H, t, *J* = 5.59 Hz), 3.11 (2H, s), 3.38 (2H, m), 5.30 (2H, m). ^13^C NMR: δ 14.1, 22.6, 25.6, 27.1, 29.5, 31.8, 36.6, 38.7, 48.4, 77.0, 129.8, 173.7.

**Figure 2:**
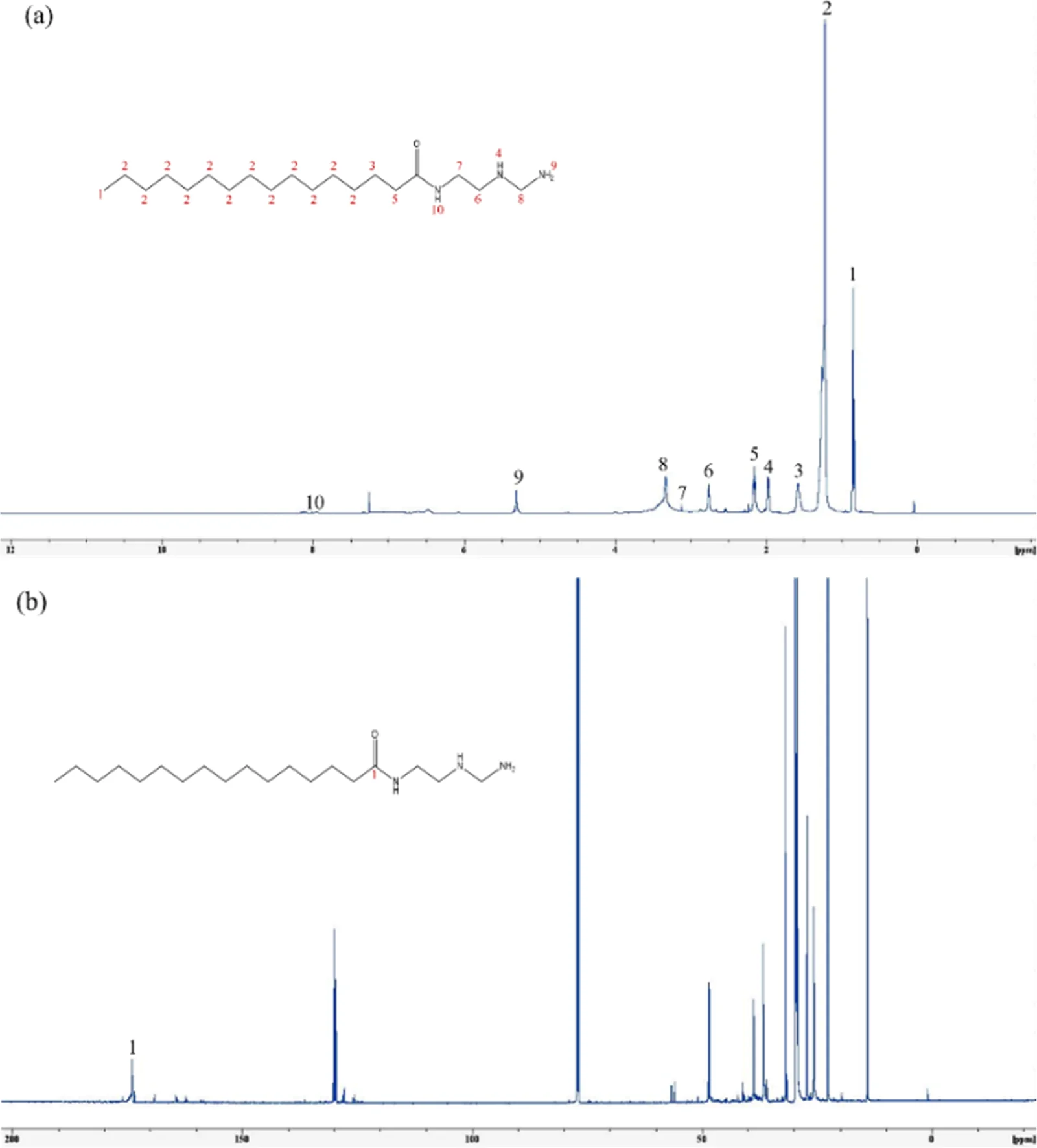
Spectra of synthetic PFAD-based amide (a) ^1^H NMR and (b) ^13^C NMR.

**Figure 3:**
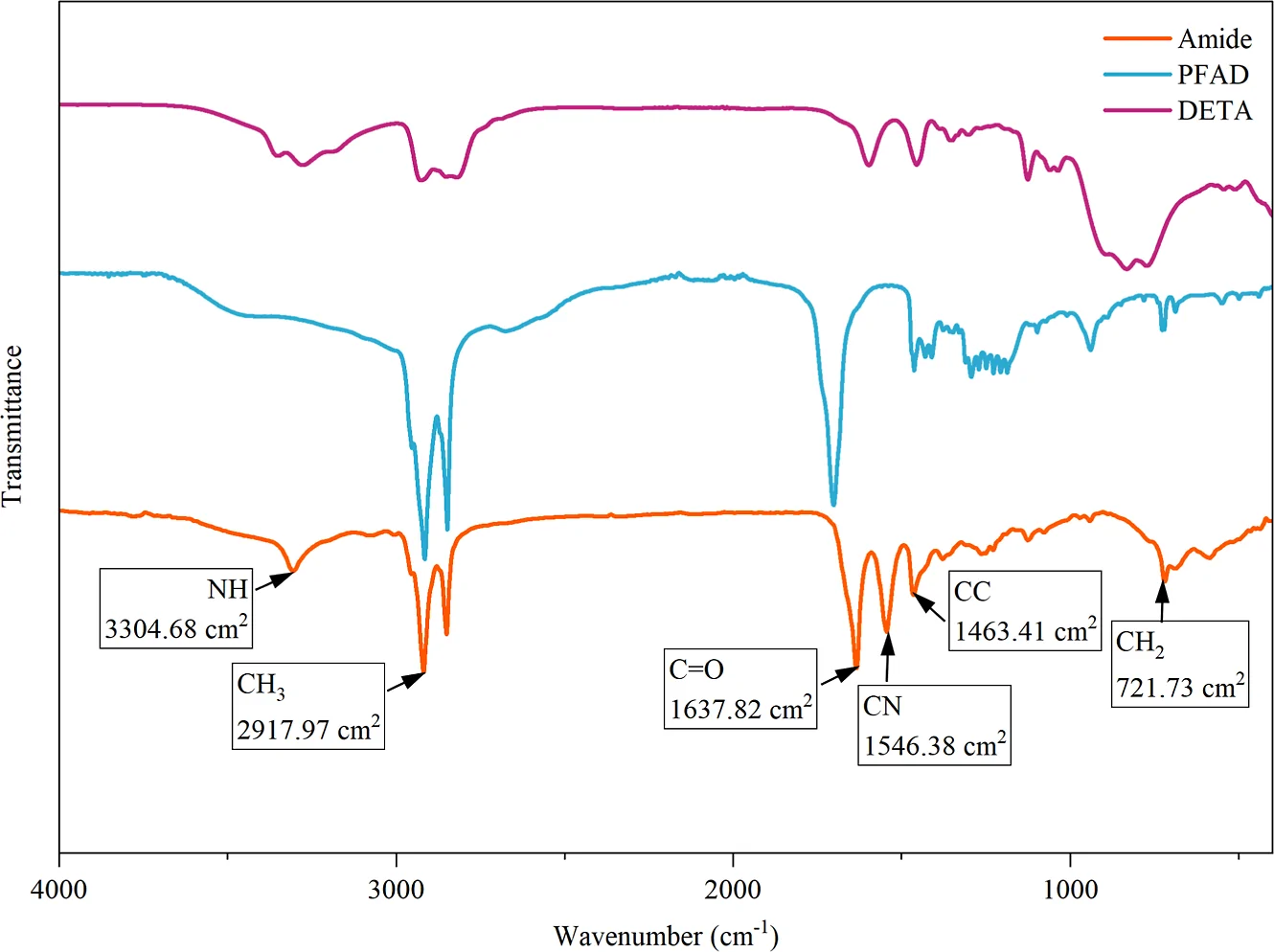
FTIR spectra of the synthetic PFAD-based amide.

In the FTIR spectrum (Figure 3), the formation of amide was suggested by the presence of signals indicating the stretching of CH_3_ and CH_2_ at 2918 cm^–1^ and 722 cm^–1^, respectively. Additionally, a peak at 3305 cm^–1^ indicated N–H stretching. The stretching of the carbonyl group (C=O) resulted in a signal at 1638 cm^–1^, while the stretching of C–N was observed at 1546 cm^–1^. Additionally, the acidity of the as-received PFAD and synthetic PFAD-based imidazoline samples was characterized using TAN and pH which were 175.08 mgKOH/g and 2.2, respectively. The synthesis of the corrosion inhibitor resulted in a change in acidity, with a decrease in acidity observed. The TAN value of synthetic PFAD-based samples was found to reduce to 7.05 mgKOH/g whereas the pH was increased to 4.8.

### 3.2 Open Circuit Potential

Open circuit potential (OCP) describes the thermodynamic behavior of metallic materials in electrochemical corrosion systems a positive OCP shift is commonly associated with a more noble surface condition or improved corrosion resistance, whereas a negative shift indicates increased electrochemical activity. Moreover, OCP measurements serve as an effective tool for evaluating the formation and stability of protective films on metal surfaces. The development of a compact and stable surface film can significantly impede electrochemical corrosion processes.^10,24^

Figure 4 illustrates the OCP behavior of X65 carbon steel in a CO_2_-saturated 1% NaCl solution with and without corrosion inhibitors at various concentrations. In general, the OCP values exhibit a decreasing trend during the initial exposure period (0–8 h), after which a stable state is achieved. For the uninhibited system, the OCP shifted from −0.6004 V to −0.6401 V within the first 8 h and subsequently remained nearly constant at approximately −0.64 V throughout the entire testing period. In contrast, in the presence of corrosion inhibitors, the OCP curves generally shifted toward more positive values, indicating enhanced corrosion resistance. An exception was observed at an inhibitor concentration of 100 ppm, where the OCP values were slightly lower than those measured in the blank solution.

**Figure 4:**
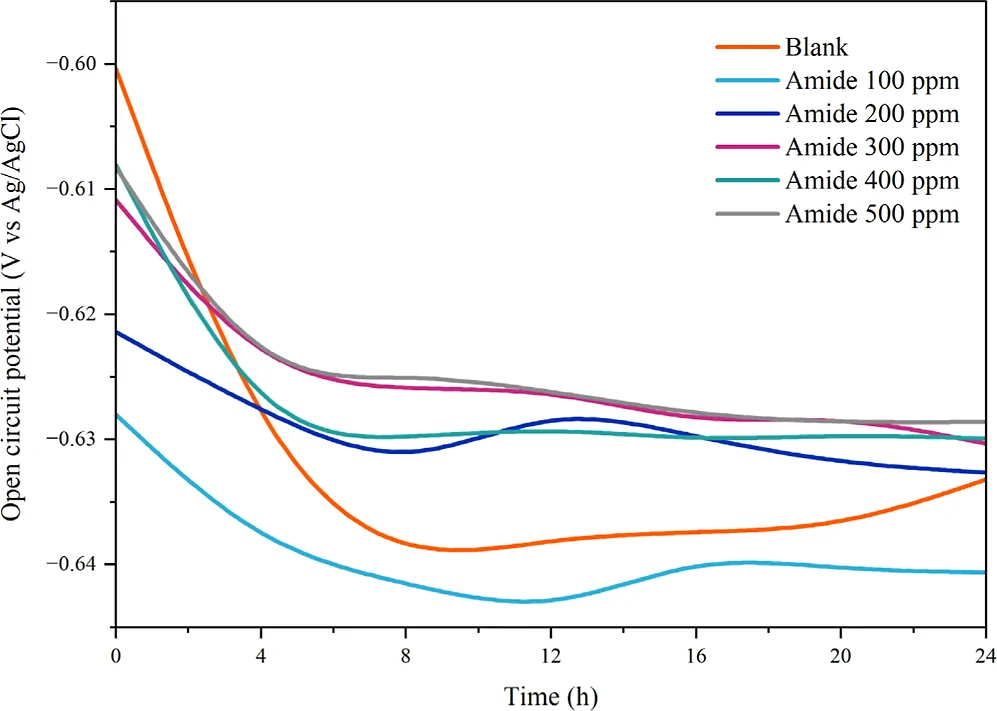
A variation of OCP of X65 carbon steel specimens in the absence and presence of different concentrations of corrosion inhibitors in CO_2_-saturated 1% NaCl solution.

Upon addition of the synthetic PFAD-based amide inhibitor at concentrations ranging from 100 to 500 ppm, the OCP values changed from −0.6367 to −0.6498 V, −0.6286 to −0.6354 V, −0.6145 to −0.6253 V, −0.6124 to −0.6340 V, and −0.6090 to −0.6269 V, respectively. The presence of the corrosion inhibitor significantly accelerated the stabilization of the electrochemical system, with steady-state OCP values being reached within approximately 6 h, compared to 8 h for the uninhibited system.

The positive shift in OCP values in the inhibited systems suggests the formation of a protective film on the steel surface, which reduces electrochemical activity and enhances corrosion resistance. Moreover, the shorter time required to attain steady-state conditions further confirms the rapid adsorption and film-forming capability of the PFAD-based corrosion inhibitor.^26^

### 3.3 Potentiodynamic Polarization and Linear Polarization Resistance Analysis

Figure 5 presents the potentiodynamic polarization curves of X65 carbon steel measured in a CO_2_-saturated 1 wt % NaCl solution after 6 h of immersion in the absence and presence of the as-prepared amide inhibitor. Electrochemical parameters such as corrosion potential (*E*_corr_), corrosion current density (*I*_corr_), anodic and cathodic Tafel slopes (β_*a*_ and β_*c*_), and inhibition efficiency (η) were derived from an extrapolation of linear Tafel regions, as summarized in Table 1. The η value can be determined using eq 1.

As observed from Figure 5 and Table 1, the introduction of the synthetic amide inhibitor significantly influenced the electrochemical behavior of X65 carbon steel. The presence of the inhibitor led to noticeable changes in both anodic and cathodic branches of the polarization curves, accompanied by a substantial reduction in corrosion current density (*I*_corr_), indicating effective corrosion inhibition in the CO_2_-containing saline environment.

**Figure 5:**
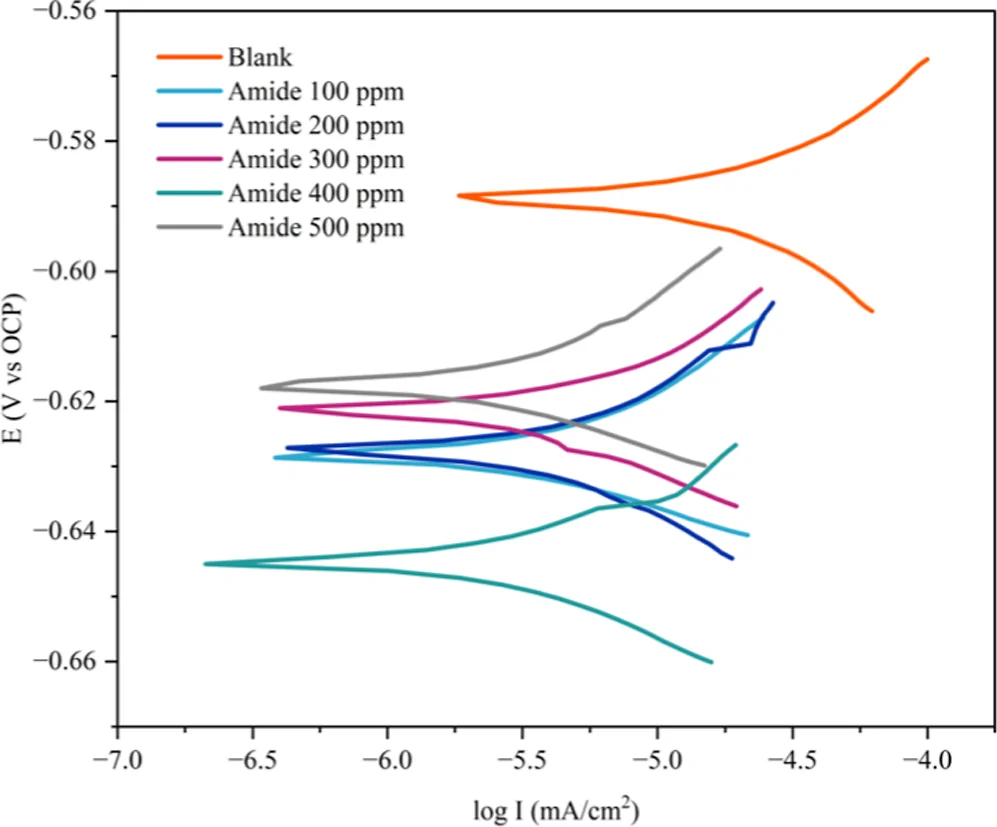
A variation of PDP of X65 carbon steel specimens in the absence and presence of different concentrations of corrosion inhibitors in CO_2_-saturated 1% NaCl solution.

**Table 1:** Polarization Parameters of X65 Carbon Steel Specimens Immersed in 1 wt % NaCl Solution in the Absence and Presence of Different Concentrations of the Synthetic PFAD-Based Amide

	PDP				LPR			
Conc. (ppm)	*E*_corr_ (V vs Ag/AgCl)	*I*_corr_ (μA/cm^2^)	*|*β_*a*_ *|* (mV/dec)	|β_*c*_| (mV/dec)	**η**_PDP_(%)	CR (mmpy)	*R*_p_ (Ω cm^2^)	_LPR_(%)
0	–0.6197	91.7840	53.0605	70.601	-	1.0939	495.4600	-
100	–0.6201	37.5103	60.5256	39.6646	59.1319	0.4241	1018.3567	51.3471
200	–0.6299	53.4713	68.5603	61.9286	41.7422	0.6460	907.2333	45.3878
300	–0.6216	30.2056	65.9776	49.0506	67.0904	0.3057	1760.1367	71.8510
400	–0.6409	39.2980	53.0383	57.7086	57.1842	0.4394	1155.7900	57.1323
500	–0.6253	35.2620	67.3230	52.1720	61.5815	0.4155	1260.1667	60.6829

#### 3.3.1 Effect of Inhibitor on Corrosion Potential

The corrosion potential (*E*_corr_) exhibited a slight shift toward more negative values after the addition of the PFAD-based amide inhibitor. This behavior is frequently reported for organic corrosion inhibitors in CO_2_-saturated NaCl environments and is primarily associated with modifications in surface electrochemical kinetics induced by inhibitor adsorption rather than changes in bulk solution chemistry.

In CO_2_-containing saline systems, the corrosion potential represents a dynamic balance between the anodic dissolution of iron and the cathodic reduction reactions involving dissolved CO_2_ species. When inhibitor molecules adsorb onto the steel surface, they alter the relative rates of these anodic and cathodic processes. The adsorption of PFAD-based amide molecules partially blocks electrochemically active sites, leading to a redistribution of surface reaction pathways and a consequent adjustment of the mixed corrosion potential.

The observed negative shift in *E*_corr_ suggests that the adsorption of inhibitor molecules slightly suppresses the anodic dissolution process to a greater extent during the early stages of film formation, while the cathodic reactions remain partially accessible through uncovered surface regions. This temporary imbalance in reaction kinetics results in a moderate displacement of the corrosion potential toward more negative values.

However, the magnitude of the potential shift (Δ*E*_corr_) remained below 85 mV relative to the uninhibited system. According to established electrochemical criteria, such a limited displacement indicates that the inhibitor does not predominantly affect only one electrochemical half-reaction. Instead, the PFAD-based amide simultaneously influences both anodic and cathodic reactions, thereby maintaining a relatively stable corrosion potential.

This behavior is characteristic of mixed-type corrosion inhibitors, in which adsorption-induced changes in surface kinetics modify the polarization behavior of both anodic and cathodic processes without causing a pronounced potential displacement. The limited shift in *E*_corr_ therefore reflects balanced inhibition of both electrochemical reactions rather than selective anodic or cathodic control.

#### 3.3.2 Reduction of Corrosion Current Density

A substantial decrease in corrosion current density *I*_corr_ was observed upon the introduction of the PFAD-based amide. The *I*_corr_ value decreased from 91.78 μA cm^–2^ for the uninhibited solution to significantly lower values in the presence of the inhibitor, demonstrating a clear reduction in the overall corrosion rate.

The reduction in *I*_corr_ can be attributed to the adsorption of inhibitor molecules onto the steel surface, forming a protective film that partially isolates the metal from the aggressive chloride-containing electrolyte. This adsorbed layer decreases the number of active corrosion sites and impedes charge transfer between the metal surface and the electrolyte, resulting in improved corrosion resistance. Although complete surface coverage may not be achieved, the inhibitor film is sufficiently stable to markedly suppress the corrosion process in the CO_2_-saturated NaCl medium.

#### 3.3.3 Influence on Anodic and Cathodic Reactions

The presence of the PFAD-based amide led to noticeable changes in both β_*a*_ and β_*c*_ Tafel slopes. Compared with the blank solution, an increase in β_*a*_ values was observed, indicating that the anodic dissolution of iron was effectively hindered by inhibitor adsorption on anodic active sites.

In contrast, β_*c*_ values exhibited a slight decrease after inhibitor addition. In CO_2_-saturated NaCl environments, the cathodic reaction is primarily associated with the reduction of carbonic acid species and dissolved CO_2_. The observed variation in β_*c*_ suggests that the inhibitor also affects cathodic kinetics, although to a lesser extent than the anodic process.

The concurrent modification of both β_*a*_ and β_*c*_ demonstrates that the PFAD-based amide influences both anodic and cathodic reactions.

#### 3.3.4 Inhibition Mechanism and Inhibitor Classification

The simultaneous changes observed in both anodic and cathodic polarization behaviors clearly indicate that the synthetic PFAD-based amide functions as a mixed-type corrosion inhibitor. This classification arises from its ability to influence the kinetics of both electrochemical half-reactions occurring at the metal–solution interface.

The inhibition behavior of the PFAD-based amide is mainly controlled by the adsorption of inhibitor molecules onto the steel surface, which is commonly observed for organic corrosion inhibitors containing heteroatoms, polar functional groups, and hydrophobic chains.^11–13,20–25,27^ The amide and amine groups in the PFAD-derived molecule contain nitrogen and oxygen atoms with lone-pair electrons. These atoms can interact with vacant d-orbitals of Fe atoms on the steel surface through donor–acceptor interactions, resulting in the formation of coordinate bonds. In addition to chemical adsorption, physical adsorption may also occur through electrostatic attraction between protonated amide/amine groups and the charged steel/electrolyte interface in chloride-containing solution. The combined effect of chemical and physical adsorption promotes surface coverage and improves the stability of the adsorbed inhibitor film under CO_2_-saturated saline conditions, as shown in Figure 6.

**Figure 6:**
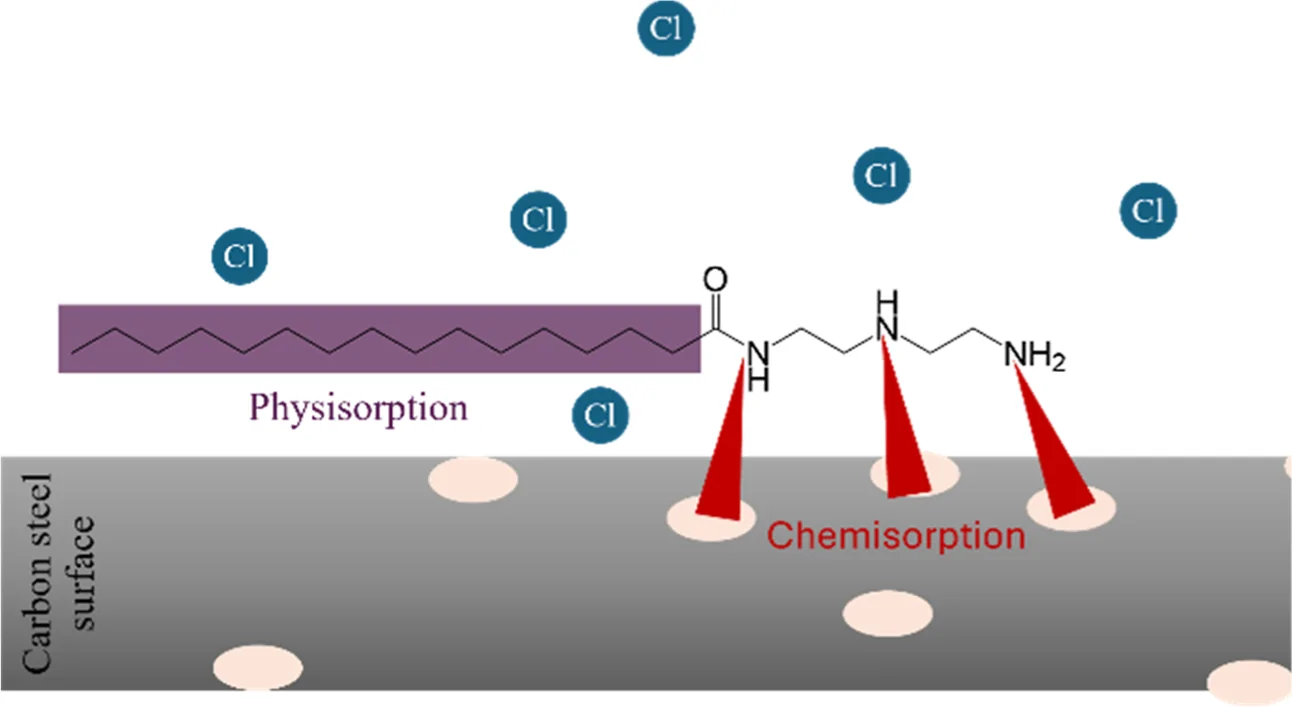
Inhibition mechanism of PFAD-based amide on X65 carbon steel in CO_2_-saturated NaCl solution.

After the polar amide/amine groups are adsorbed on the steel surface, the long hydrophobic hydrocarbon chains derived from PFAD are oriented outward from the metal surface. This molecular arrangement forms a compact hydrophobic protective layer that reduces the diffusion of aggressive species, including Cl^–^, dissolved CO_2_, and water molecules, toward the steel substrate. Consequently, both anodic steel attack and cathodic reduction reactions are hindered, leading to improved corrosion protection, as illustrated in Figure 6.

The suppression of anodic reactions results from the blockage of active dissolution sites, while the inhibition of cathodic processes occurs due to restricted electron transfer and reduced accessibility of reactants at the metal surface. As a result, the overall corrosion rate decreases substantially without inducing a dominant polarization of either reaction. Because the inhibitor affects both anodic and cathodic processes in a relatively balanced manner, no significant displacement of the corrosion potential is observed. This explains the limited shift in *E*_corr_ (<85 mV) recorded in the polarization measurements and further confirms the mixed-type inhibition behavior of the PFAD-based amide.

Overall, the corrosion inhibition performance is controlled by adsorption-driven film formation, in which the synergistic contribution of the polar amide functional groups and the hydrophobic PFAD chains produces an effective protective layer capable of mitigating corrosion in CO_2_-saturated NaCl environments.

#### 3.3.5 Effect of Inhibitor Concentration

The inhibition efficiency exhibited a clear dependence on inhibitor concentration, reflecting the adsorption-controlled nature of the corrosion inhibition process. Maximum inhibition performance was achieved at an inhibitor concentration of 300 ppm, yielding efficiencies of 67.09% and 71.85% as determined from PDP and LPR measurements, respectively.

At lower inhibitor concentrations (100–200 ppm), the limited availability of inhibitor molecules in the electrolyte restricts the extent of adsorption onto the steel surface. Under these conditions, surface coverage remains incomplete, leaving a significant number of electrochemically active sites exposed to the corrosive environment. Consequently, both anodic dissolution and cathodic reduction reactions continue to occur on uncovered regions, resulting in relatively lower inhibition efficiencies.

As the inhibitor concentration increases, a greater number of PFAD-based amide molecules become available for adsorption, promoting the formation of a more continuous protective film. At approximately 300 ppm, adsorption equilibrium is approached, and surface coverage reaches an optimal level. At this concentration, the inhibitor molecules are able to arrange themselves effectively on the metal surface, forming a compact and stable adsorption layer that maximally suppresses charge transfer and limits electrolyte access to the substrate. However, further increases in inhibitor concentration beyond this optimal value (400–500 ppm) did not lead to proportional improvements in corrosion protection. Instead, a slight decrease in inhibition efficiency was observed. This behavior can be attributed to several surface-related phenomena, including molecular aggregation in solution, competitive adsorption between inhibitor molecules, and steric hindrance at the metal–solution interface. Excess inhibitor molecules may disrupt the ordered arrangement of the adsorbed layer, resulting in a less compact or nonuniform film. Additionally, at high concentrations, repulsive interactions between adsorbed species can reduce adsorption stability and promote partial desorption, thereby diminishing the effectiveness of the protective barrier. As a result, the corrosion inhibition efficiency no longer increases monotonically with inhibitor concentration.

These findings demonstrate that the corrosion inhibition performance of the synthetic PFAD-based amide is governed by surface adsorption dynamics rather than bulk concentration alone. The existence of an optimum concentration at 300 ppm highlights the importance of achieving balanced surface coverage and molecular organization to form a stable and effective inhibitor film under the present experimental conditions.

The inhibition efficiencies calculated from PDP (η_PDP_) and LPR (η_LPR_) measurements exhibited consistent trends across all inhibitor concentrations, confirming the reliability of the electrochemical data. PDP analysis provides detailed insight into corrosion mechanisms by evaluating anodic and cathodic kinetics over a wide potential range. However, this technique may partially disturb the inhibitor film during polarization. In contrast, LPR measurements are conducted within a narrow potential window around *E*_corr_, minimizing surface perturbation and offering more representative corrosion rate values under near-equilibrium conditions.

The close agreement between PDP and LPR derived efficiencies indicates that the synthetic PFAD-based amide provides stable and reproducible corrosion inhibition in CO_2_-saturated NaCl solution.

Figure 7 illustrates the variation in corrosion rate of X65 carbon steel measured at 40 min intervals over a total immersion period of 6 h in CO_2_-saturated 1 wt % NaCl solution, in the absence and presence of different concentrations of the synthetic PFAD-based amide inhibitor. For all tested conditions, the corrosion rate exhibited a rapid decrease during the initial 60–80 min of immersion, followed by a gradual reduction throughout the remaining exposure period. The sharp decline observed at the early stage can be attributed to the initial interaction between the steel surface and the electrolyte, during which corrosion products and inhibitor molecules progressively adsorb onto the metal surface. As immersion proceeds, the corrosion process becomes increasingly controlled by surface film formation, resulting in a slight decrease in corrosion rate.

**Figure 7:**
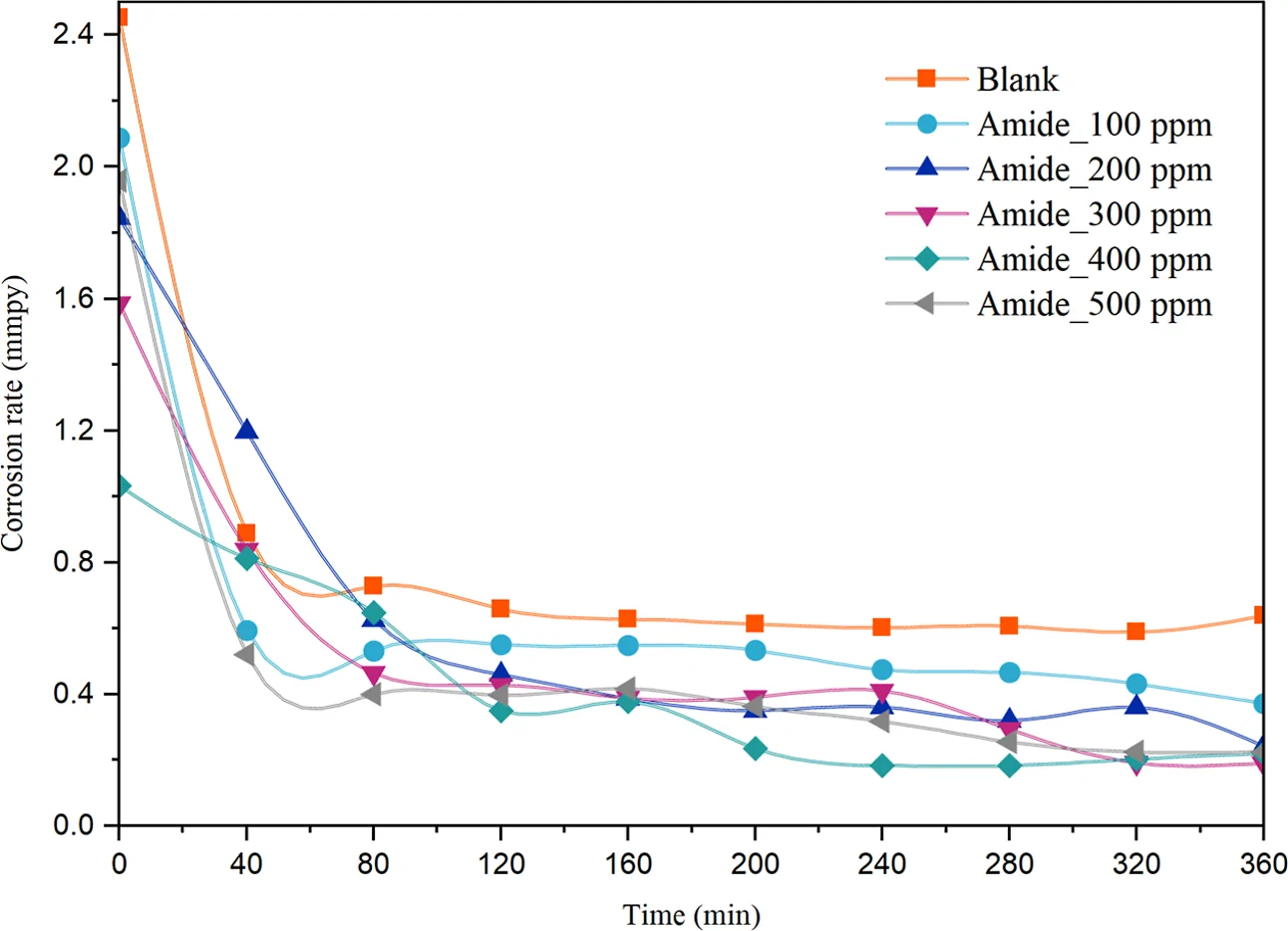
Variation of corrosion rate of X65 carbon steel specimens as a function of immersion time in the absence and presence of different concentrations of the synthetic PFAD-based amide inhibitor in CO_2_-saturated 1 wt % NaCl solution.

The presence of the corrosion inhibitor significantly reduced both the initial corrosion rate (at 0 min) and the overall corrosion rate during the entire immersion period. This behavior indicates that the synthetic PFAD-based amide effectively retards the corrosion process by forming an adsorbed protective layer that limits the electrochemical activity of the steel surface. After 6 h of immersion, the corrosion rates were determined to be 0.4241, 0.6460, 0.3057, 0.4394, and 0.4155 mmpy at inhibitor concentrations of 100, 200, 300, 400, and 500 ppm, respectively. In contrast, the uninhibited system exhibited a considerably higher corrosion rate of 1.0939 mmpy, confirming the beneficial effect of the inhibitor under CO_2_-saturated saline conditions.

The variation in corrosion rate with inhibitor concentration is consistent with the electrochemical results obtained from PDP and LPR measurements. At low inhibitor concentrations (100–200 ppm), the amount of inhibitor molecules available for adsorption is insufficient to achieve complete surface coverage. As a result, the protective film remains discontinuous, allowing corrosion reactions to proceed at exposed active sites, which explains the relatively higher corrosion rates observed at these concentrations. At an inhibitor concentration of 300 ppm, the lowest corrosion rate was recorded. This concentration provides an optimal balance between inhibitor availability and surface adsorption, enabling the formation of a compact and stable protective film. The enhanced surface coverage effectively suppresses both anodic metal dissolution and cathodic reduction reactions, leading to the highest corrosion inhibition performance. At higher inhibitor concentrations (400–500 ppm), the corrosion rate increased slightly compared with the optimum condition. This behavior may be attributed to the formation of less uniform inhibitor layers due to molecular aggregation, competitive adsorption, or excessive film thickness. Such phenomena can induce film instability, partial detachment, or self-delamination of the adsorbed layer, thereby reducing its protective effectiveness despite the higher inhibitor dosage.

The observed dependence of corrosion rate on inhibitor concentration indicates that the thickness, density, and stability of the inhibitor film are strongly influenced by the amount of synthetic PFAD-based amide present in solution, in agreement with previously reported adsorption-controlled inhibition mechanisms.^27^

Overall, the corrosion rate trends obtained from long-term immersion measurements are in good agreement with the results of the polarization studies. The combined PDP, LPR, and time-dependent corrosion rate analyses clearly demonstrate that the synthetic PFAD-based amide acts as an effective corrosion inhibitor for X65 carbon steel in CO_2_-saturated NaCl environments, with optimal performance achieved at an inhibitor concentration of 300 ppm.

### 3.4 Electrochemical Impedance Spectroscopy

EIS measurement is used to study the behaviors of electrochemical corrosion reactions. The results can be used to create an equivalent circuit model to simulate the physical characteristics occurring on the metal surface. Figure 7a shows a Nyquist plot, a graph depicting the relationship between real and imaginary impedance. The plot appears as a semicircle, indicating the charge transfer process. A larger semicircle signifies a higher charge transfer resistance, whereas a smaller semicircle indicates lower corrosion resistance. In this study, X65 carbon steel was exposed to different inhibitor concentrations (0, 100, 200, 300, 400, 500 ppm) within the same environmental conditions for 24 h. The results show that at inhibitor concentrations of 400 and 500 ppm, the steel demonstrated the highest charge transfer resistance. This increase in charge transfer resistance indicates that the inhibitor is effectively reducing the corrosion rate of the steel, suggesting improved corrosion resistance at these concentrations. The higher the charge transfer resistance, the more difficult it becomes for corrosion to occur, which is a positive outcome, indicating that the inhibitor is functioning well at these concentrations to protect the steel from corrosion. Corresponding to Table 2, the further parameters are as follows. *R*_s_ represents the solution resistance, *R*_f_ represents the inhibitor film resistance, *R*_ct_ represents the charge transfer resistance, and *R*_p_ represents total resistance (*R*_f_ + *R*_ct_). The Constant Phase Element (CPE) is widely used to model the electrical behavior of electrodes in electrochemical systems, particularly in the study of corrosion. In corrosion systems, the surface of metals or electrodes is rarely smooth or homogeneous. Instead, it often exhibits irregularities, roughness, or other imperfections. These surface characteristics can significantly influence the electrochemical response, making the electrical behavior of the electrode more complex than that of an ideal capacitor. As corrosion progresses, the surface of the metal may undergo chemical and physical changes, including the formation of corrosion products or protective films. These films can alter the way the electrode interacts with the electrolyte, affecting its impedance. The formation of a protective layer, for instance, can reduce the electrochemical activity at the electrode surface, leading to an increase in the impedance at low frequencies. When corrosion inhibitors are introduced into the system, their effect on the metal surface is often to promote the formation of a protective film that prevents further corrosion. This protective layer can be organic, inorganic, or a combination of both, and it acts as a barrier to ions and electrons, reducing the rate of corrosion. The presence of such inhibitors can lead to noticeable changes in the impedance behavior of the system, which are captured by variations in the CPE parameters. Specifically, corrosion inhibitors often lead to modifications in the CPE parameters such as the capacitance (*Q*) and the exponent (*n*). The capacitance *Q* reflects the ability of the electrode surface to store charge, and it can change due to the formation of the protective film, which modifies the surface’s electrochemical properties. The exponent *n* is indicative of the degree of deviation from ideal capacitive behavior. The value of *n* ranges between 0 and 1.^27^ When *n* is 1, CPE functions as a capacitor. If *n* is 0, CPE can be considered as a resistor. When *n* is −1, CPE behaves like an inductance. In a corrosion system, a value of *n* less than 1 suggests nonideal behavior due to surface irregularities or inhomogeneities. The inhibitor-induced formation of a protective layer might cause the surface to become more uniform, leading to a shift in *n* toward 1, indicating a more ideal capacitive response. These changes in CPE parameters serve as an indication that the corrosion inhibitor has altered the physicochemical properties of the surface, particularly the composition and structure of the protective film. By analyzing these modifications, it is possible to gain insights into the effectiveness of the inhibitor and how it influences the protective layer, ultimately helping to assess its ability to prevent or reduce corrosion.

**Table 2:** Impedance Parameters of X65 Carbon Steel Immersed in 1 wt % NaCl Solution in the Absence and Presence of Different Concentrations of Synthetic PFAD-Based Amide

Conc. (ppm)	*R*_s_ (Ω cm^2^)	*R*_f_ (Ω cm^2^)	CPE1		*R*_ct_ (Ω cm^2^)	CPE2		*R*_p_	*X* ^2^	**η**_**EIS**_(%)
			*Y*_0_	*n*		*Y*_0_	*n*			
0^a^	41.8820	22.4730	2.00 × 10^–8^	0.9929	63.2220	2.34 × 10^–3^	0.8735	85.695	0.0024	-
100	52.4725	-	-	-	77.4520	2.16 × 10^–3^	0.8743	77.4520	0.0039	–10.64 ± 19.84
200	49.7390	33.8140	1.25 × 10^–3^	0.5067	185.9200	1.01 × 10^–3^	0.9050	219.734	0.0277	61.00 ± 10.65
300	46.6305	47.6305	7.28 × 10^–4^	0.5318	289.4300	6.65 × 10^–4^	0.9008	337.0830	0.0245	74.58 ± 1.19
400	48.6100	89.2040	8.54 × 10^–4^	0.4485	384.9450	4.76 × 10^–4^	0.9404	474.1490	0.0296	81.93 ± 1.89
500	44.2665	91.4635	8.05 × 10^–4^	0.4367	383.8750	4.91 × 10^–4^	0.9285	475.3385	0.0391	81.97 ± 2.06

a In the case of the blank solution (0 ppm), the impedance response does not exhibit distinct film resistance (*R*_f_) and charge transfer resistance (*R*_ct_). Instead, the obtained resistance reflects the overall resistance of carbon steel, which behaves as a porous material. This behavior is attributed to the absence of a protective inhibitor film on the metal surface, resulting in nonuniform electrochemical activity and electrolyte penetration through surface defects and corrosion sites.

In this study, the characterization of the protective film using the CPE model reveals an *n* value ranging from 0.4 to 0.5. This indicates that ion diffusion plays a significant role in the electrochemical process, suggesting that the protective film formed by the synthetic PFAD-based amide is not fully effective in preventing corrosion. The *n* value in this range typically suggests nonideal behavior, meaning the film’s protective capacity is still developing and may not yet fully block the diffusion of corrosive ions. On the other hand, for the metal surface itself, the CPE’s *n* value approaches 1, which is indicative of a more uniform and stable surface with less variation in its electrochemical properties. This suggests that, while the protective film is not fully formed, it still offers a degree of protection against corrosion, possibly by slowing down or reducing the rate of corrosion on the metal surface. While the synthetic PFAD-based amide may not yet provide complete protection, the formation of a protective film does improve the corrosion resistance of the metal surface, although further development of the film may be required to achieve optimal protection.

The data obtained from Table 2 can be used to calculate the corrosion inhibition efficiency from eq 3. The observed inhibition efficiencies for the inhibitor dosages of 100, 200, 300, 400, and 500 ppm were −10.64%, 61.00%, 74.58%, 81.93%, and 81.97% respectively. According to Figure 7, in the system containing a 100 ppm concentration of corrosion inhibitor, the observed increase in corrosion potential, compared to the system without an inhibitor, suggests that the inhibitor concentration is not sufficient to form an effective protective film. A protective film is crucial in shielding the metal surface from corrosive agents, and an inadequate concentration of the inhibitor may fail to adequately adsorb onto the surface or form a stable, effective barrier. As a result, the metal surface may still be vulnerable to corrosion, leading to an elevated corrosion potential. Furthermore, the acidic nature of the inhibitor can contribute to an increase in corrosion. A lower pH accelerates corrosion by increasing the availability of hydrogen ions (H^+^), which can react with the metal surface, leading to a more aggressive corrosion process. Thus, the combined effects of an inadequate inhibitor concentration and the inhibitor’s acidic properties could explain the observed increase in corrosion potential and the negative impact on corrosion inhibition efficiency. As the inhibitor concentration increases (e.g., 200 ppm and above), the inhibition efficiency improves, suggesting that higher dosages of the inhibitor are more effective at forming a protective film and reducing corrosion.

Figure 8b illustrates the Bode plot of impedance magnitude (|Z|) as a function of frequency for blank and inhibited system. The curve behavior across different frequency regions provides critical insights into the corrosion protection efficiency of the amide inhibitor.

**Figure 8:**
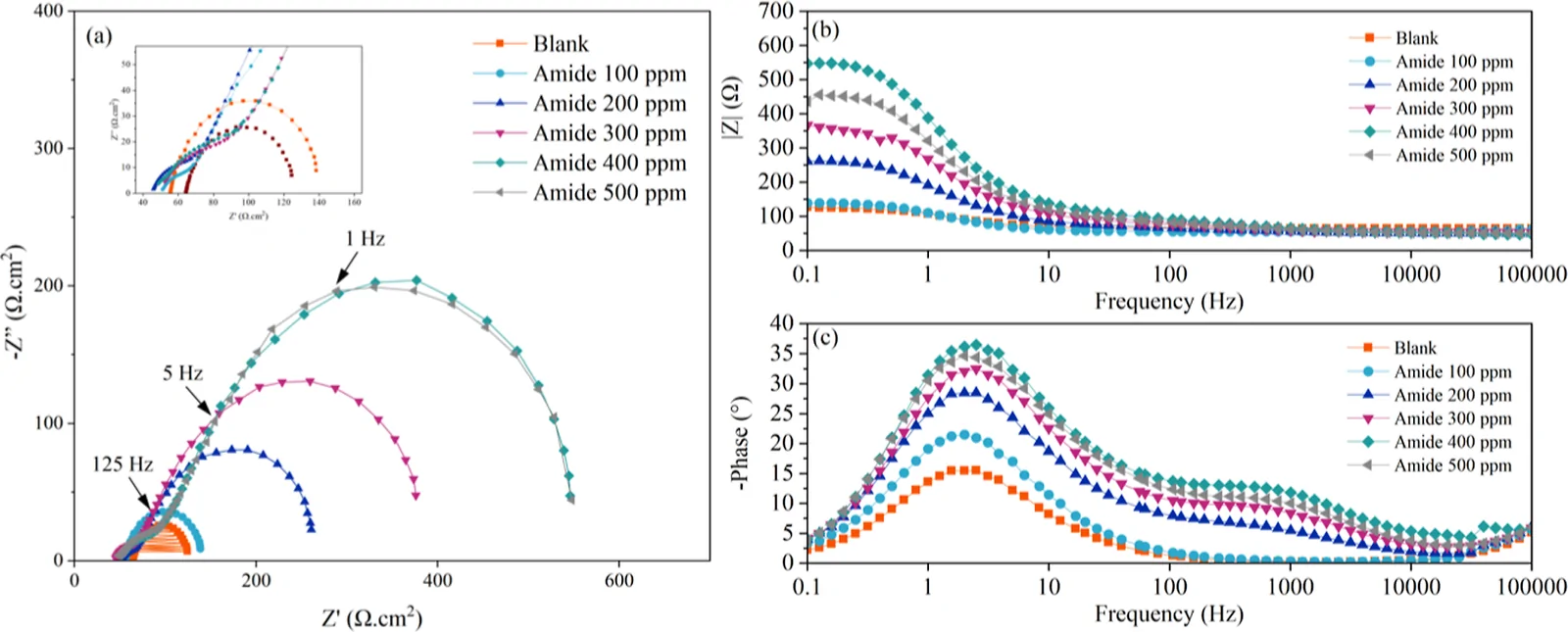
A variation of (a) Nyquist and Bode plot; (b) Impedance Magnitude and (c) Phase angle of X65 carbon steel immersed in CO_2_-saturated 1% NaCl solution in the absence and presence of different concentrations of PFAD-based inhibitor.

At high frequencies (>10 kHz), all curves converge to similar *|Z|* values, corresponding to the solution resistance, *R*_s_. In this region, electrochemical reactions cannot occur rapidly enough to influence impedance, and thus *R*_s_ dominates the response. The negligible difference among samples here confirms that the electrolyte and base resistance remain unchanged by the inhibitor. In the midfrequency range (approximately 1–100 Hz), the impedance is primarily governed by the film resistance (*R*_f_), which represents the resistance offered by the protective amide film. A higher *R*_f_ suggests a more robust, intact inhibitor layer that effectively hinders current flow. As seen in Figure 8b, the *|Z|* values rise progressively with increasing amide concentration up to 400 ppm, with the 400 ppm sample exhibiting the highest |Z| in this region. This indicates the formation of a highly resistive protective film that minimizes electron and ion transfer, thus enhancing corrosion resistance. The slope in this region also reflects capacitive behavior, characteristic of a well-developed passive layer. At low frequencies (<1 Hz), the impedance is predominantly influenced by charge transfer resistance (*R*_ct_), a critical indicator of the metal’s electrochemical activity. Higher *R*_ct_ (and thus higher *|Z|*) reflects slower corrosion kinetics, implying greater resistance to charge transfer across the metal–solution interface. As shown, the 400 ppm sample again displays the highest impedance, confirming that this dosage provides optimal protection by suppressing electrochemical reactions on the metal surface. In contrast, the blank and lower-concentration of amide inhibitors (100–200 ppm) exhibit lower *|Z|* values, suggesting insufficient surface coverage or degraded inhibitor films, which permit more active corrosion.

Interestingly, a slight drop in *|Z|* at 500 ppm implies that excessive inhibitor dosage may lead to film destabilization, such as aggregation, cracking, or oversaturation, reducing the protective efficiency. This behavior highlights the importance of optimizing inhibitor concentration to maximize performance.

Figure 8c illustrates the phase angle response across a range of frequencies for varying concentrations of the amide inhibitor, offering valuable insights into the electrochemical behavior and the nature of the surface film formed. Notably, the phase angle increases with inhibitor concentration up to 400 ppm, indicating an enhancement in capacitive behavior. This trend suggests the development of a compact, protective passive film that effectively impedes charge transfer processes.

The sample with 400 ppm inhibitor displays the highest phase angle peak and the widest frequency span of elevated phase angles, reflecting superior barrier characteristics. This behavior is typically associated with the formation of a uniform, adherent, and highly dielectric film layer, which contributes to enhanced corrosion protection. However, a slight reduction in the peak phase angle observed at 500 ppm implies a decline in film performance, possibly due to overadsorption or micelle formation that disrupts the uniformity and integrity of the protective layer.

At midto-high frequencies, the phase angle approaches 0°, indicating a predominantly resistive response, which can be attributed to *R*_s_ and *R*_f_. In the high-frequency region, the phase angle ranges between 0° and −40°, signifying a combination of resistive and capacitive behavior. This suggests the presence of a semiprotective film that exhibits partial charge storage capability.

Overall, the changes in phase angle with frequency and concentration confirm that the electrochemical system transitions from resistive to mixed resistive-capacitive behavior depending on the inhibitor dose. The marked improvement in capacitive response at 400 ppm highlights this concentration as optimal for forming a stable and effective passive film that suppresses metal dissolution.

Table 2 presents the EIS parameters for X65 carbon steel immersed in 1 wt % NaCl solution with and without various concentrations of synthetic PFAD-based amide inhibitor. These parameters offer insight into the corrosion inhibition performance and film-forming ability of the inhibitor at each concentration.

At 100 ppm, the system shows negligible improvement in polarization resistance (*R*_p_ = 77.4520 Ω·cm^2^) and low charge transfer resistance (*R*_ct_ = 77.4520 Ω·cm^2^), coupled with a negative inhibition efficiency (η_***EIS***_ = −10.64%). This indicates insufficient inhibitor concentration to form a stable passive film. Correspondingly, Figure 9a illustrates the equivalent circuit model for this condition, consisting of *R*_s_, *R*_f_, and a single CPE. This model represents a mostly unprotected surface with no distinct film layer.

**Figure 9:**
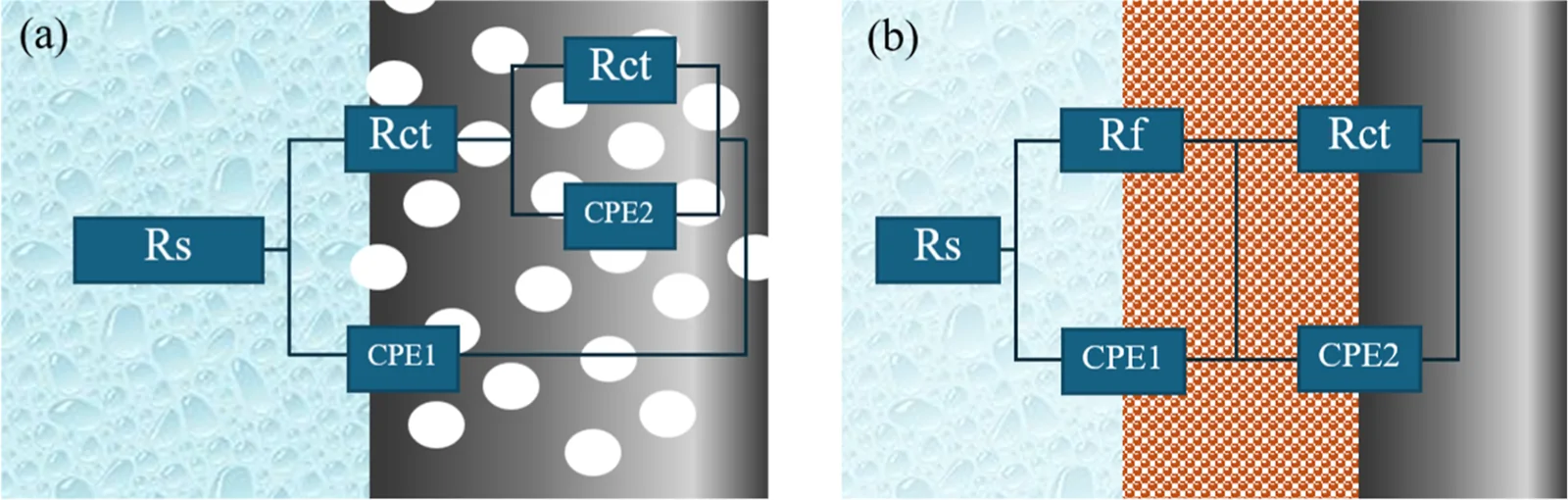
Equivalent circuit diagrams used to fit impedance data in the absence of synthetic PFAD-based amide (a) and presence of synthetic PFAD-based amide (b).

In contrast, at concentrations ≥200 ppm, the data demonstrate a significant increase in both *R*_ct_ and *R*_p_, indicating improved barrier properties due to the formation of a protective film. For instance, at 200 ppm, *R*_p_ increases to 219.73 Ω·cm^2^, and *R*_ct_ to 185.92 Ω·cm^2^, with η_**EIS**_ jumping to 61.00%. This trend continues, peaking at 400 ppm, where *R*_p_ reaches 474.15 Ω·cm^2^ and *R*_ct_ is 384.95 Ω·cm^2^—the highest values recorded—accompanied by the highest inhibition efficiency (η_**EIS**_ = 81.93%). This signifies optimal film formation and corrosion protection at this concentration. Although 500 ppm also shows high *R*_p_ (475.34 Ω·cm^2^) and η_**EIS**_ (81.97%), the slightly lower *R*_ct_ compared to 400 ppm (383.88 Ω·cm^2^) may suggest film saturation or slight degradation due to excess inhibitor, which could hinder uniform film formation. The equivalent circuit for these higher concentrations, illustrated in Figure 9b, includes two *R*_f_ -CPE branches in parallel with *R*_s_. This model accounts for both the solution resistance and the dual-layer film resistance, reflecting the complex nature of the passive film and its capacitive behavior.

The inclusion of dual CPEs in Figure 9b is indicative of the layered structure of the protective film formed at higher inhibitor concentrations. These elements represent nonideal capacitive behavior, often associated with surface heterogeneities and the distributed nature of the protective layer.

### 3.5 Surface Morphology

The SEM images of X65 carbon steel after 24 h exposure to CO_2_-saturated 1 wt % NaCl solution are shown in Figure 10. The specimen in the absence of inhibitor (Figure 10a) exhibited a severely damaged and highly roughened surface with nonuniform corrosion products and localized pits. These features indicate continuous corrosive attack on the steel surface by the chloride-containing electrolyte under CO_2_-saturated conditions.

**Figure 10:**
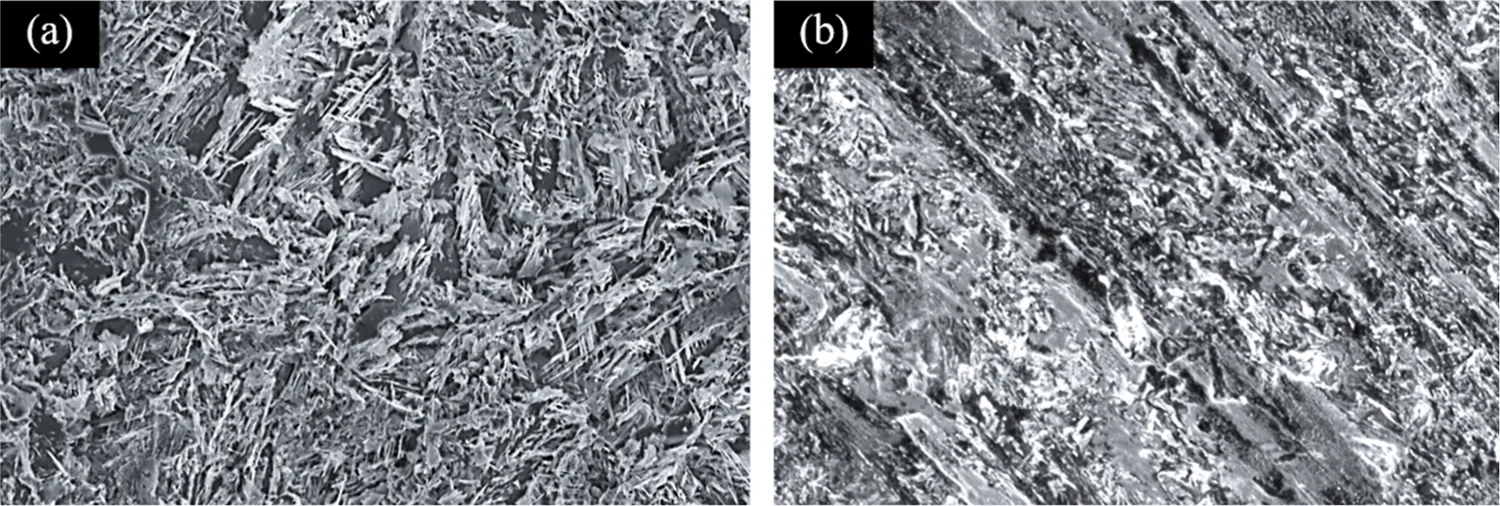
Surface morphology of X65 carbon steel after 24 h immersion in CO_2_-saturated 1 wt % NaCl solution: (a) the absence of synthetic PFAD-based amide (b) presence of synthetic PFAD-based.

In contrast, the specimen exposed to the PFAD-based amide inhibitor (Figure 10b) displayed a comparatively more compact and uniform surface. The visible corrosion damage was less pronounced than that observed on the uninhibited specimen. This morphological improvement suggests that PFAD-based amide molecules adsorbed onto the steel surface and reduced direct contact between the steel substrate and aggressive species such as Cl^–^, dissolved CO_2_, and water molecules.

For the purpose of elucidating the adsorption behavior of the synthetic amide on the X65 carbon steel surface, FTIR was performed on the extracted adsorbed film after corrosion exposure. The steel specimen was immersed in a CO_2_-saturated 1% NaCl solution containing 500 ppm of synthetic amide for 24 h. The FTIR spectra of the extracted surface film are presented in Figure 11 and compared with that of the pure synthetic amide shown in Figure 3. Notably, the characteristic C–O stretching vibration observed in the pure inhibitor spectrum completely disappeared after adsorption onto the steel surface. This disappearance suggests the direct involvement of oxygen-containing functional groups in the adsorption process, likely through coordination interactions with the metal surface. In addition, the absorption bands corresponding to the CH_3_ stretching and C–N vibrations became more intense and exhibited noticeable shifts to 2924 cm^–1^ and 1546 cm^–1^, respectively. These spectral shifts indicate changes in the electronic environment of the functional groups following adsorption, confirming the interaction between the nitrogen-containing moieties of the synthetic amide and the X65 carbon steel surface.

**Figure 11:**
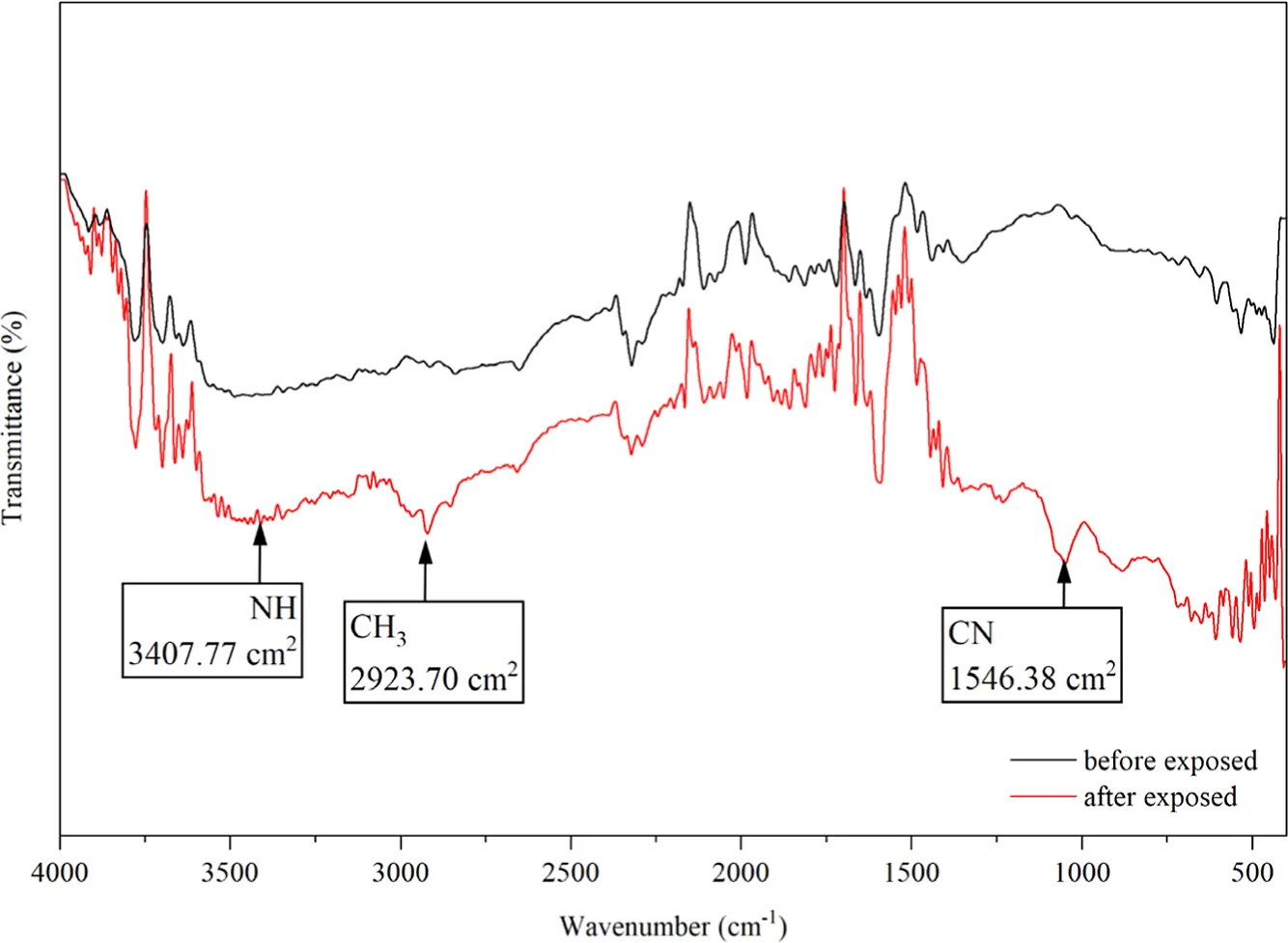
FTIR spectra of the adsorbed inhibitor film formed on X65 carbon steel after immersion in a CO_2_-saturated 1 wt % NaCl solution containing 500 ppm of the synthetic PFAD-based amide for 24 h.

Overall, the observed peak disappearance, intensity enhancement, and wavenumber shifts provide strong evidence for the formation of an adsorbed protective film (inhibitor–metal complex) on the steel surface, supporting the proposed corrosion inhibition mechanism of the synthetic amide in CO_2_-saturated saline media.

## 4 Conclusions

A PFAD-based amide corrosion inhibitor was successfully synthesized using PFAD and DETA as starting materials. The formation of the amide structure was confirmed through ^1^H NMR, ^13^C NMR, and FTIR spectroscopic analyses.

The corrosion inhibition performance of the synthesized amide toward X65 carbon steel in CO_2_-saturated 1 wt % NaCl solution was evaluated using LPR, PDP, and EIS at different inhibitor concentrations. Based on the experimental results, the main conclusions can be summarized as follows:

- After 6 h of immersion, the presence of the synthetic PFAD-based amide led to an increase in the anodic Tafel slope (β_*a*_) and a decrease in the cathodic Tafel slope (β_*c*_), accompanied by a significant reduction in corrosion current density compared with the uninhibited solution. These changes indicate that the inhibitor suppresses both anodic metal dissolution and cathodic reactions, confirming its mixed-type inhibition behavior. The highest inhibition efficiency of 67.09% was achieved at an inhibitor concentration of 300 ppm.
- Within the same 6 h immersion period, the addition of the synthetic amide markedly decreased the corrosion rate relative to the blank solution. The maximum inhibition efficiency determined from corrosion rate measurements was 71.85% at a concentration of 300 ppm.
- After 24 h of exposure, the inhibitor concentrations of 400 and 500 ppm exhibited comparable inhibition efficiencies of 81.93% and 81.97%, respectively, representing the highest protection levels observed throughout the testing period.

Overall, the PFAD-based amide demonstrated stable and effective corrosion inhibition performance in CO_2_-saturated saline environments, attributable to the formation of an adsorbed protective film on the steel surface. These findings highlight the strong potential of PFAD-derived amide compounds as environmentally benign corrosion inhibitors for carbon steel applications in CO_2_-containing oil and gas systems.
